# The Role of Extracellular Vesicles in Osteoporosis: A Scoping Review

**DOI:** 10.3390/membranes12030324

**Published:** 2022-03-14

**Authors:** Weifei Zhang, Pengzhou Huang, Jianjing Lin, Hui Zeng

**Affiliations:** 1 Department of Bone & Joint Surgery/National & Local Joint Engineering Research Center of Orthopaedic Biomaterials, Peking University Shenzhen Hospital, Shenzhen 518036, China; zhangweifei@bjmu.edu.cn; 2 National Cancer Center & Shenzhen Hospital, Chinese Academy of Medical Sciences and Peking Union Medical College, Shenzhen 518116, China; 13pzhuang@stu.edu.cn; 3 Arthritis Clinical and Research Center, Peking University People’s Hospital, Beijing 100044, China; 4 Department of Sports Medicine and Rehabilitation, Peking University Shenzhen Hospital, Shenzhen 518036, China

**Keywords:** extracellular, vesicles, osteoporosis

## Abstract

As an insidious metabolic bone disease, osteoporosis plagues the world, with high incidence rates. Patients with osteoporosis are prone to falls and becoming disabled, and their cone fractures and hip fractures are very serious, so the diagnosis and treatment of osteoporosis is very urgent. Extracellular vesicles (EVs) are particles secreted from cells to the outside of the cell and they are wrapped in a bilayer of phospholipids. According to the size of the particles, they can be divided into three categories, namely exosomes, microvesicles, and apoptotic bodies. The diameter of exosomes is 30–150 nm, the diameter of microvesicles is 100–1000 nm, and the diameter of apoptotic bodies is about 50–5000 nm. EVs play an important role in various biological process and diseases including osteoporosis. In this review, the role of EVs in osteoporosis is systematically reviewed and some insights for the prevention and treatment of osteoporosis are provided.

## 1. Introduction

Osteoporosis is defined as a systemic bone disease characterized by bone loss and bone microstructure destruction, and the diagnostic criteria for osteoporosis is a bone mineral density T score of less than −2.5 [1]. Osteoporosis is a common disease among the elderly, with high incidence and serious complications, and about 40% of 50-year-old female patients will have osteoporotic fractures for the rest of their life [2]. The most serious complication of osteoporosis is osteoporotic fractures, which reduces the quality of life of elderly patients with osteoporosis and increases their mortality [3]. RANK/RANKL/OPG signaling pathway plays a pivotal role in the maturation of osteoclasts and bone remodeling, and RANK/RANKL interaction allows osteoclast maturation, thereby promoting osteoporosis occurrence. In addition, the WNT signaling pathway and PTH signaling pathways are also involved in the occurrence of osteoporosis [4,5,6,7,8]. Bisphosphonates are widely used in the clinical treatment of osteoporosis. Although bisphosphonates have satisfactory therapeutic effects, they also have some side effects. Therefore, new prevention and treatment methods for osteoporosis are critical [9,10,11,12].

EVs are secreted by cells to exchange information between cells, and contain proteins, lipids, and nucleic acids [13]. EVs can be divided into three types based on their diameter and biological behavior, namely exosomes, microvesicles, and apoptotic bodies. The diameter of exosomes is 30–150 nm, that of microvesicles is 100–1000 nm, and that of apoptotic bodies is about 50–5000 nm [14]. EVs play an important role in various biological processes, including cell communication, apoptosis, immune response, and tumor development [15]. For example, glioblastoma can promote the proliferation and treatment resistance of surviving tumor cells by secreting EVs [16]. EVs are also involved in the progression of various diseases, such as tumors and immune diseases. Some researchers have reported that CD8 T cells suppress the immune response by releasing EVs containing CD73 to promote adenosine production when being activated [17]

Similarly, EVs are also important in the occurrence and development of osteoporosis. Hui Xie et al. found that EVs play a key role in osteoporosis, and the EVs of human-urine-derived stem cells are enriched in CTHRC1 and OPG, which can effectively promote osteogenesis, inhibit osteoclasts, and prevent osteoporosis [18]. In addition, many researchers have discovered that EVs play a key role in the cause, diagnosis and treatment of osteoporosis, and that engineered EVs also play an important role in the treatment of osteoporosis [19,20,21,22]. Given that EVs play a profound role in osteoporosis, we chose to systematically review their effects. The role of EVs in osteoporosis can be summarized by three aspects: the potential role of EVs in the etiology of osteoporosis, and their potential diagnostic and therapeutic effects. Some clues for the prevention and treatment of osteoporosis were provided.

## 2. Production and Release of EVs

**Figure 1:**
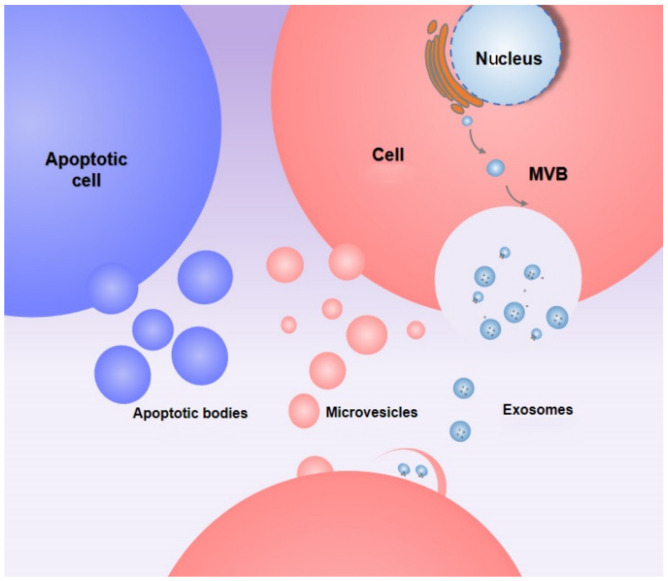
Biogenesis of different EV subtypes.

EVs can be divided into apoptotic bodies, microvesicles, and exosomes (Figure 1). Apoptotic bodies are produced by shedding cells during apoptosis, while microvesicles (MVs) are produced by the plasma membrane by budding—the production process of MVs is related to the increase of calcium ion concentration in the cytoplasm— and exosomes are secreted by polycysts and released mainly through exocytosis, which is associated with cytoskeleton activation and calcium ion concentration [14]. It is currently believed that the production of exosomes involves two pathways: the endosomal sorting complex required for transport (ESCRT)-dependent pathway and the ESCRT-independent pathway. The specific mechanism of the ESCRT-dependent pathway is that the cell membrane invaginates to form early endosomes, and then multivesicular endosomes (MVBs) are formed. MVBs with high cholesterol content fuse with the cytoplasmic membrane and are released into the extracellular matrix to form exosomes, while MVBs with low cholesterol content are degraded by the lysosomal pathway. The ESCRT-independent pathway for exosome production mainly involves ceramides, ceramides induce the formation of luminal vesicles, and it promotes the entry of biomolecules rich in proteins and RNAs into luminal vesicles, and finally after the fusion of the luminal vesicles with the cell membrane, the exosomes release [23,24].

## 3. The Potential Role of EVs in the Etiology of Osteoporosis

EVs are secreted by cells to the outside of cells that are involved in the occurrence and development of many diseases. For example, a report from *CELL* pointed out that Bloodstream African trypanosomes can produce EVs to cause anemia [25]. EVs can also play a role in the occurrence and development of osteoporosis. Dawei Liu et al. proposed that the reduction in the formation of apoptotic bodies significantly impairs the self-renewal and osteogenic and adipose differentiation of bone marrow mesenchymal stem cells. Specifically, the apoptotic bodies secreted by bone marrow mesenchymal stem cells contain RNF146 and miR-328-3p, and the two molecules can inhibit Axin1, thereby activating the Wnt/β-catenin pathway. Therefore, their study determined the previously unknown role of EVs in bone homeostasis, and the potential use of the EVs in the treatment of osteoporosis was found [21]. In addition, Rongyao Xu et al. concluded that the expression of miR-31a-5p in the exosomes of BMSCs of old rats was increased, and these BMSCs with high expression of miR-31a-5p showed increased adipogenic differentiation capacity and decreased osteogenic differentiation capacity. Moreover, the inhibition of exosomes miR-31a-5p can prevent bone loss and reduce osteoclast activity in old rats. Therefore, a conclusion was drawn that the exosomes of mesenchymal stem cells involved in the occurrence of osteoporosis and provided potential osteoporosis treatment value [26]. Many researchers have confirmed that EVs affect the occurrence and development of osteoporosis. Sylvia Weilner et al. illustrated that senescent endothelial cells secreted microvesicle contaning miR-31 and that these vesicles inhibit the osteogenic differentiation of mesenchymal stem cells, Perrine J Martin et al. demonstrated that adipogenic RNAs are transferred in osteoblasts via bone marrow adipocyte-derived EVs [27,28].

## 4. EVs as Potential Diagnostic Tool in Osteoporosis

EVs contain proteins, lipids, mRNA, and ncRNAs, and the characteristics of EVs determine that they are suitable as disease markers. According to Daniel Liu et al., plasma EVs contain unannotated small RNA clusters, so they are suitable to be biomarkers for the detection of early hepatocellular carcinoma. Likewise, the abnormal content of EVs may could be used as the markers of age-related osteoporosis [22,29,30,31] (Table 1).

**Table 1:** EVs as potential diagnostic tool in osteoporosis.

Moleculars	Regulated	Samples (Experimental Group-Control Group)	*p* Value	Reference
Vinculin et al.	upregulated	28-28	<0.05	[32]
PSMB9 et al.	upregulated	60-60	<0.001	[33]
miR-4746-3p et al.	down-regulated	12-6	0.000487	[34]
tRF-25 et al.	upregulated	40-40	<0.05	[35]

Chunhui Huo et al., analyzed different protein profiles of microvesicles in the serum of normal subjects, osteopenia patients, and osteoporosis patients. A total of about 200 differentially expressed proteins were identified and quantified from the serum. Compared with the normal group, in the osteopenia group and the osteoporosis group, 19 proteins were up-regulated and 5 proteins were down-regulated. Then, they selected three candidate proteins for preliminary verification, including Vinculin, Filamin A, and Profilin 1. Profilin 1 was further prevalidated in independent sample sets, which could be distinguished between the osteoporosis group, osteopenia group, and normal group (*p* < 0.05). Their data indicated that the serum microvesicles proteome can be used for evaluation and diagnosis as important indicators of bone-loss disease [32]. In addition, Ming Chen and others from China 301 Hospital compared the protein profiles of plasma exosomes from 60 patients with osteoporosis, osteopenia, and normal bone mass, aiming to find potential new diagnoses. They found 45 differentially expressed proteins, four of which, namely PSMB9, AARS, PCBP2, and VSIR, were further verified. Based on these results, they constructed an exosomal protein index to compare individuals with osteoporosis for nonosteoporotic individual classification, and the AUC for classification performance evaluation is 0.805 [33].

Noncoding RNAs in EVs can also be used as potential markers of osteoporosis. In order to evaluate the EVs as potential diagnostic tool in osteoporosis of exosomal microRNA (miRNA) on osteoporosis in menopausal women, Jian-Li Shao and others recruited 6 menopausal women without osteoporosis and 12 menopausal women with osteoporosis, then isolated their serum exosomes, and detected their miRNA expression by miRNA high-throughput sequencing. The results revealed that 191 abnormal miRNAs were found in the osteoporosis group of menopausal women, of which 72 of them were up-regulated and 121 were down-regulated. They concluded that abnormal serum exosomal miRNAs are related to osteoporosis in menopausal women as a biomarker [34].

In addition, some researchers have discovered that transfer RNA-derived fragments (tRFs) are also abnormally expressed in the plasma of osteoporosis patients. Yan Zhang et al. used centrifugation to collect plasma-derived exosomes from 40 healthy controls and 40 osteoporosis patients, and detected tRF in plasma exosomes by small RNA sequencing, and finally found that 11 up-regulated tRF and 18 down-regulated tRF were identified in osteoporosis, compared with normal controls. Later, they further confirmed that the RF-25-R9ODMJ6B26 (tRF-25), tRF-38-QB1MK8YUBS68BFD2 (tRF-38) and tRF-18-BS68BFD2 (tRF-18) of plasma exosomes have higher expression level in osteoporosis, so plasma exosomes tRF-25, tRF-38 and tRF-18 may have satisfactory diagnostic value in osteoporosis [35].

In terms of diagnostic tools, because osteoporosis is caused by a variety of factors, it is difficult to think that a single component can predict osteoporosis. As a result, risk score systems that incorporate diverse proteins, lipids, mRNA, and ncRNAs found in EVs might be necessary to be used as potential diagnostic tool in osteoporosis, although there is currently no such risk score system, it is hoped that there will be a standard risk score system for EVs in the diagnosis of osteoporosis in the future, providing a new method for the diagnosis of osteoporosis.

## 5. The Potential Therapeutic Effects of EVs in Osteoporosis

**Figure 2:**
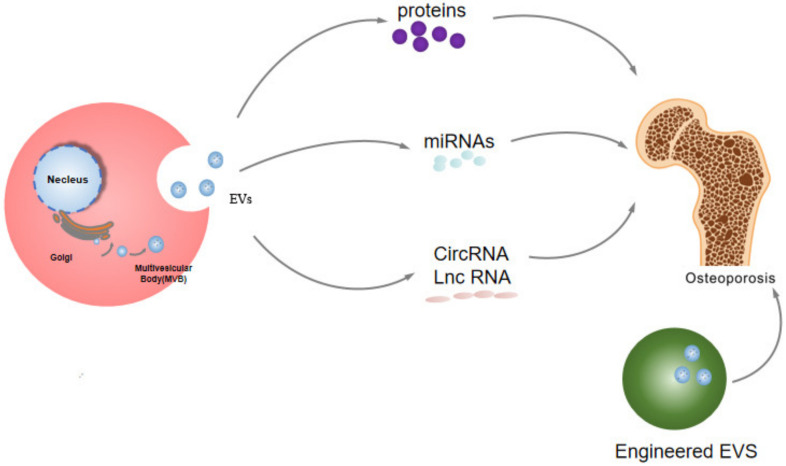
The potential therapeutic effects of EVs in osteoporosis.

Many researchers hold the view that proteins and nucleic acids in EVs have potential therapeutic value (Figure 2), including various diseases such as tumors, immune diseases, inflammation and cardiovascular diseases, etc. [36,37,38,39,40,41,42,43,44,45]. For instance, Naohiro Seo et al. proposed that activated CD8+ T cells from healthy mice release cytotoxic EVs causing marked attenuation of tumor invasion and metastasis by apoptotic depletion of mesenchymal tumor stromal cells [46]. Lingling Jiang et al. supported EVs with TGF-β1-dependent immunosuppressive activity are produced by intestinal epithelial cells (IECs) under physiological conditions, they can decrease IBD severity [47].

Kian F Eichholz et al. demonstrated that osteocytes can be mechanically activated to secrete EVs to regulate mesenchymal stem cell differentiation [48]. EVs not only play a therapeutic role in other diseases, but also play an important role in osteoporosis [28,48,49,50,51,52,53,54,55,56]. Based on Xin Qi et al., exosomes secreted by human-induced pluripotent stem cell-derived mesenchymal stem cells repair critical-sized bone defects through enhanced angiogenesis and osteogenesis in osteoporotic rats [57]. Lili Deng et al. provided the information that imipramine can prevent bone loss by inhibiting osteoblast-derived microvesicles [58]. Therefore, EVs have potential therapeutic value in osteoporosis. EVs contain protein and noncoding RNA, so the potential therapeutic effects of EVs in osteoporosis were introduced from four aspects: protein, miRNA, lncRNA, and circRNA.

In addition, engineered EVs also play a potential role in the treatment of osteoporosis.

### 5.1. The Potential Therapeutic Effects of Proteins of EVs in Osteoporosis

As a very common component in EVs, protein has many functions, including promoting transcription and protein–protein interactions. Meanwhile, it also plays an important role in various life activities, and has potential therapeutic effects on osteoporosis (Table 2) [59,60,61].

**Table 2:** The role of EV-associated proteins in osteoporosis.

Proteins	Source	Regulated	Functions	Reference
CTHRC1/OPG	EVs	upregulated	inhibit osteoporosis	[9]
RNF146	apoptotic body	upregulated	inhibit osteoporosis	[21]
CLEC11A	EVs	upregulated	inhibit osteoporosis	[62]
NLRP3	exosomes	upregulated	inhibit osteoporosis	[63]
WNT1/WNT5A/WNT7A/WNT9A	EVs	down	inhibit osteoporosis	[64]
OPG	EVs	upregulated	inhibit osteoporosis	[65]

Chun-Yuan Chen et al. collected EVs from human-urine-derived stem cells (USG), and then injected these EVs into a mouse model of osteoporosis, which proved that these EVs can enhance bone formation and inhibit osteoclast resorption; the specific mechanism is the enrichment of CTHRC1 and OPG proteins are necessary for inducing bone formation and inhibiting osteoclast resorption. Therefore, USC-EVs can be said to be a very promising method of treatment for osteoporosis [9]. Dawei Liu et al. demonstrated that RNF146 in circulating apoptotic bodies can treat osteoporosis [21]. In addition, Yin Hu et al. revealed that the increased expression of CLEC11A in EVs of human umbilical cord mesenchymal stromal cells can promote the transition from adipogenesis to osteogenic differentiation, thereby inhibiting osteoporosis, which may represent the prevention and treatment of osteoporosis (Potential Drugs for Symptoms) [62]. Based on Lei Zhang et al., the NLRP3 inflammasome in the exosomes of adipose-derived mesenchymal stem cells can inhibit osteoporosis in rats [63]. According to Kyoung Soo Lee et al., the therapeutic effects of EVs were derived from the treatment of adipose tissue-derived stem cells (ASC-EVs) on osteoporosis. On the other hand, they found that osteoprotegerin is highly enriched in ASC-EV by transmission electron microscopy, dynamic light scattering, zeta potential, flow cytometry, cytokine arrays, enzyme-linked immunoassays and adsorption assays. The intravenous injection of ASC-EV can reduce bone loss in osteoporotic mice, while OPG-depleted ASC-EVs did not show anti-osteoclastogenesis effects, which indicated that OPG is very important for the therapeutic effect of ASC-EVs. Their research showed that ASC-EVs are very promising as acellular therapeutics for the treatment of osteoporosis. All in all, many studies have proved that the protein in EVs plays a significant role in the progress of osteoporosis with potential therapeutic value for osteoporosis [64,65].

### 5.2. MiRNA

MiRNAs in EVs play an important role in osteoporosis (Table 3). The mechanism of the action of miRNA is generally to inhibit the translation of the target genes or to degrade them by binding to them, and miRNAs in EVs have therapeutic potential for osteoporosis [22]. Sylvia Weilner et al. analyzed the plasma in osteoporosis and normal human plasma. The level of miR-31 in the plasma of the elderly and osteoporosis patients is elevated in microvesicles, which can inhibit osteogenic differentiation by targeting Frizzled-3. They knocked down miR-31 and found that the osteogenic ability of mesenchymal stem cells was enhanced and the phenotype of osteoporosis was reduced. Therefore, they discovered that miR-31 is a potential treatment for osteoporosis [27]. LB Jiang et al. extracted the exosomes of mesenchymal stem cells from healthy subjects and osteoporosis patients, and found that the expression of microRNA-21 in exosomes increased, which confirmed that microRNA-21 can bind to SMAD7, and the expression of SMAD7 decreased. MicroRNA-21 inhibits the osteogenic differentiation of mesenchymal stem cells and promotes the occurrence and development of osteoporosis [66]. Hongyuan Song et al. performed miRNA sequencing on the vascular endothelial cell exosomes of normal mice and osteoporotic mice, and the sequencing results showed that the expression of miR-155 was much higher. The blockade of miR-155 level reversed the inhibition of EC-Exos on osteoclast induction, which confirmed that exosomal miR-155 may have the potential to treat osteoporosis [67].

**Table 3:** The role of EV-associated miRNA in osteoporosis.

Gene	Source	Regulated	Functions	Reference
miR-31	microvesicles	upregulated	promote osteoporosis	[27]
miR-21	exosomes	upregulated	promote osteoporosis	[66]
miR-328-3P	apoptotic bodies	upregulated	inhibit osteoporosis	[21]
miR-155	exosomes	upregulated	inhibit osteoporosis	[67]
miR-3960	EVs	upregulated	inhibit osteoporosis	[20]
miR-22-3p	EVs	down	promote osteoporosis	[68]
miR-214-3p	exosomes	down	promote osteoporosis	[69]
miR-186	exosomes	upregulated	inhibit osteoporosis	[70]
miR-29b-3p	EVs	down	inhibit osteoporosis	[71]
miR-143/145	EVs	upregulated	promote osteoporosis	[72]
miR-139-5p	exosomes	upregulated	promote osteoporosis	[73]
miR-935	exosomes	upregulated	inhibit osteoporosis	[74]
miR-424-5p	exosomes	upregulated	promote osteoporosis	[75]
miRNA-19b-3p	exosomes	upregulated	inhibit osteoporosis	[76]
miR-27a-5p	EVs	upregulated	inhibit osteoporosis	[77]
miR-27a	EVs	upregulated	inhibit osteoporosis	[78]

Apoptotic bodies are also a type of EVs. Dawei Liu et al., concluded that miR-328-3p in the apoptotic bodies of mesenchymal stem cells inhibits Axin1, it activates the wnt/β-catenin pathway and promotes osteogenesis and inhibits osteoporosis, which implies the potential use of apoptotic bodies in the treatment of osteoporosis [21]. Xueliang Zhang et al., provided the information that in the ovariectomized mouse model, the expression of miR-22-3p in the exosomes of bone marrow mesenchymal stem cells was inhibited, and the overexpression of miR-22-3p increased the alkaline phosphatase (ALP) activity and activity of matrix mineralization of bone marrow mesenchymal stem cells. The specific mechanism is that miR-22-3p targets fat mass and obesity-associated gene (FTO) and inhibits the expression of FTO, and FTO inhibition inactivates the MYC/PI3K/AKT pathway, thereby enhancing the osteogenic differentiation in vivo and in vitro. The conclusion proved that the source of mesenchymal stem cells MiR-22-3p delivered by EVs can be used as a potential treatment for osteoporosis. In addition, there are many confirmations that miRNAs in EVs have therapeutic potential in osteoporosis [20,68,69,70,71,72,73,74,75,76,77,78].

Therefore, we know that miRNAs of EVs play a huge role in the treatment of osteoporosis, and many core mechanisms of EVs for the treatment of osteoporosis are realized through miRNAs, they can change the direction of osteogenic and adipogenic differentiation of mesenchymal stem cells to treat osteoporosis, and they can also directly change the pathological process of osteoporosis.

### 5.3. lncRNA and circRNA

The lncRNA and circRNA in EVs also play a role in the progression of osteoporosis (Table 4).

Recently, accumulating evidence has demonstrated that ncRNAs could be efficiently delivered to recipient cells using EVs as a carrier, and therefore can exert a critical role in musculoskeletal diseases including osteoporosis [79,80]. Xucheng Yang et al. tested the exosomes of mesenchymal stem cells, and found that the expression in the cells of MALAT1 increased in exosomes of mesenchymal stem cells, and MALAT1 cancer promote the osteogenic differentiation of mesenchymal stem cells, because it can be used as a sponge of miR-34c to promote the expression of SATB2. The authors also conducted a rescue experiment: MiR -34c reversed the effects of MALAT1, and SATB2 reversed the effects of miR-34c in ovariectomized mice. Therefore, it can be concluded that MALAT1 in mesenchymal stem cell exosomes can promote the osteogenic differentiation of mesenchymal stem cells as a new method for the treatment of osteoporosis [81].

As a type of RNA molecule with a closed-loop structure, circular RNA plays an important role in various life activities, and it also contain circular RNA with potential therapeutic value for osteoporosis [79,82,83,84]. Guijun Cao et al. found that the expression of circ-Rtn4 in the exosomes of bone marrow mesenchymal stem cells increased, and the overexpression of circ-Rtn4 attenuated the cytotoxicity and apoptosis of MC3T3-E1 cells induced by TNF-α. The specific mechanism is the sponge molecule of miR-146a, and circ-Rtn4 relieves the effects of miR-146a. Circ-Rtn4 in the exosomes of bone marrow mesenchymal stem cells promotes osteogenesis and inhibits osteoporosis as a treatment for osteoporosis method [85].

**Table 4:** The role of EV-associated lncRNA and circRNA in osteoporosis.

Gene	Source	Regulated	Functions	Reference
MALAT1	exosomes	upregulated	inhibit osteoporosis	[81]
circRNA Rtn4	exosomes	upregulated	inhibit osteoporosis	[85]

### 5.4. The Role of Engineered EVs in the Treatment of Osteoporosis

Engineered EVs refer to the artificially modified EVs, with great potential in the treatment of various diseases, including tumors, spinal cord injury, inflammation, cardiovascular diseases, etc. At present, there are two methods to achieve this: one is to modify the cells, co-incubate or transfect the genes or drugs so that the genes or drugs can enter into the cells, and then collect the EVs of the cells; and another is to directly add RNAs or drugs to the EVs to make the EVs have therapeutic effects [86,87,88,89,90,91,92,93,94,95,96].

Similarly, engineered EVs also have potential therapeutic effects in osteoporosis (Table 5). Yue Zhu et al. revealed that the magnetic hydroxyapatite (MHA) scaffold can change the exosomal content of osteoclasts and promote the proliferation of osteoblasts in the osteoporosis model. The specific mechanism is that under the stimulation of the MHA scaffold, certain proteins (including ubiquitin, ATP, and reactive oxygen species) in cell-derived exosomes are reduced, while Rho kinase is increased. Rho signaling is an important regulator of osteoblast growth. Osteoblasts absorb exosomes that have a large amount of Rho signaling, and modified exosomes are beneficial to activate the Rho signaling pathway in osteoblasts, thus accelerating the proliferation of osteoblasts, and promoting osteogenesis to inhibit the occurrence and development of osteoporosis [97]. Yayu Wang et al. used click chemistry to combine EVs produced by stem cells with alendronate, and tested the hydroxyapatite affinity of Ale-EV by flow cytometry. It was obvious that Ale-EVs and hydroxyapatite have high affinity in vitro, and the bone targeting of Ale-EV was tested by fluorescence imaging in vitro. The in vitro data indicated that Ale-EVs-DiD-treated mice induced strong fluorescence in bone tissue, and the WST-8 assay reagent tested the function of Ale -EVs to promote the proliferation of mMSCs. The alkaline phosphatase test was used to detect the ability of Ale-EVs to promote the differentiation of mouse mesenchymal stem cells in vitro. The results suggested that Ale-EVs promote the growth and differentiation of mouse MSCs. All in all, the conclusion is that Ale-EVs have high affinity to bones, with great clinical application potential in the treatment of osteoporosis and low systemic toxicity [98].

There are also the overexpression of genes into cells, which indirectly obtains the engineered EVs. Ba Huang et al. transfected GPNMB overexpressing lentiviral vector and control virus into bone marrow mesenchymal stem cells, then extracted EVs rich in GPNMB to act on bone marrow mesenchymal stem cells, and analyzed the effects of GPNMB-EVs on bone marrow mesenchymal stem cells by CCK8, cytochemical staining, western blotting and RT-qPCR analysis. The results were that GPNMB-EVs significantly promoted the bone marrow mesenchyme and the proliferation of stem cells, and GPNMB-EVs activated Wnt/β-catenin signals to stimulate the osteogenesis of BMSCs, which indicated that GPNMB-EVs have broad potential as a cell-free therapy for osteoporosis [99]. Wei Liu et al. overexpressed miR-20a in bone marrow mesenchymal stem cells, and later discovered that miR-20a was successfully high expressed in exosomes of bone marrow mesenchymal stem cell. These exosomes were co-cultured with human bone marrow mesenchymal stem cells, and then they detected the proliferation, migration, and osteogenic differentiation of human bone marrow mesenchymal stem cells by CCK-8 determination, alkaline phosphatase staining, alizarin red staining, qRT-PCR and western blotting. The results proved that sEV-20a can promote the migration and osteogenesis of hBM-MSCs. In vivo, sEV-20a promotes the osseointegration in a rat model of osteoporosis, and the specific mechanism is that miR-20a can enhance osteogenesis by targeting BAMBI [100].

The engineered EVs of the above two pathways are the most common engineered EVs. Of course, there are other special ways of engineered EVs. Xiaoshan Yang et al. first produced T cell-depleting nanoparticles (TDNs) and then extracted their EVs. They discovered that these EVs can alleviate the osteogenic defects and osteopenic phenotypes of BMMSCs [101]. In addition, some researchers have revealed that many other engineered EVs have the potential value of osteoporosis [102].

**Table 5:** The role of engineered EVs in osteoporosis.

Material	Source	Assembly Method	Functions	Reference
Magnetic hydroxyapatite	exosomes	MHA stimulation	inhibit osteoporosis	[97]
alendronate	EVs	Assembly of drugs into EVs	inhibit osteoporosis	[98]
GPNMB-EVs	EVs	Lentiviral transfected cells	inhibit osteoporosis	[99]
sEV-20a	EVs	Transfection into EVs	inhibit osteoporosis	[100]
T cell-depleting nanoparticles	EVs	Extract EVs	inhibit osteoporosis	[101]
alendronic acid	EVs	biomimicking polymer vesicle	inhibit osteoporosis	[102]

## 6. Clinical Progress and Future Prospects of EVs in Osteoporosis

EVs also have clinical applications in osteoporosis, Bodo C Melnik et al. found that continuous exposure of humans to exosomes of pasteurized milk may confer a substantial risk for the development of chronic diseases of civilization including osteoporosis [103]. There are some clinical applications of extracellular vesicles in osteoporosis, it is believed that EVs will play a huge role in the diagnosis and treatment of osteoporosis in the near future.

## 7. Limitations and Coping Strategies of EVs

Although EVs show great potential in the diagnosis and treatment of osteoporosis, EVs also have certain limitations, these include large-scale production and isolation of EVs, long-term storage, in vivo stability, tissue-specific targeting and delivery strategies.

Since there are a little EVs produced by cells in the natural state, more EVs can be isolated by new high-efficiency separation methods. Traditional methods of EVs isolation include ultracentrifugation, gradient ultracentrifugation, co-precipitation, size-exclusion chromatography and field flow fractionation, of which ultracentrifugation is the most commonly used method, some new separation methods could improve the separation efficiency of EVs including microfluidic filtering, contact-free sorting, and immunoaffinity enrichment [14].

There are several ways to increase the large-scale production and isolation of engineered EVs. The first is to use plasmids or viruses with the gene to transfect cells and collect the EVs [104], the second method is to extrude the EVs in an extruder after mixing with the drug [105]. Although these methods can increase a certain amount of EVs, they are still far from large-scale production and isolation; it is hoped that there will be more methods of large-scale production and isolation in the future.

The long-term storage and in vivo stability of EVs has always been difficult issues for EVs. Temperature, storage time, and freeze–thaw cycles are important factors that affect the storage stability of EVs, storage of EVs at −80 °C is a good method, the longer the storage time and the more freeze–thaw cycles, the less stable of the EVs, so reducing the storage time of the extracellular vesicles and the number of freeze–thaw cycles are also very important, in addition, freeze-drying is a promising method for the storage of EVs, and is still under investigation [106].

The tissue-specific targeting and delivery of EVs are also limiting factors for the application of extracellular vesicles. Naturally occurring EVs can reach specific places to play their roles due to their unique cell surface molecules, but EVs are difficult to target to a certain tissue or organ [107].

For in vivo stability and strategies for tissue-specific targeting of EVs, there are three approaches. The first is mother cell transformation, the principle of which is to genetically transform parent cells with EVs secretion ability to express the target protein on the cell membrane surface, and then display the target protein on the surface of the EVs membrane [108]. The second is noncovalent binding, which is to bind specific substances to the EVs membrane through noncovalent bonds, including classical interactions and hydrophobic interactions [109]. The third is covalent bonding, which is to bind specific substances to the EVs membrane through covalent bonds. Compared to noncovalent bonds, covalent bonds have higher bond energy, for example, Gang Jia et al. conjugated the EVs membrane with neuropilin-1-targeted peptide (RGERPPR, RGE) by click chemistry [110].

## 8. Conclusions

EVs have potential roles in the etiology, diagnosis and treatment of osteoporosis, and may be the key to the prevention and treatment of osteoporosis in the future. Osteoporosis is a chronic disease affecting the world with high incidence, and cone fractures and hip fractures caused by osteoporosis seriously affect the life span and quality of life of patients. EVs are particles secreted by cells and wrapped in protein and nucleic acid in a layer of lipid molecules. They play a profound role in various life activities, and EVs also play a role in osteoporosis. In this review, the potential diagnostic and therapeutic effects of EVs on osteoporosis were systematically reviewed, as well as the role of engineered EVs in osteoporosis, and a reference for the diagnosis and treatment of osteoporosis was provided.

## Data Availability

Not applicable.
